# Macrophage microRNAs integrating lipid metabolism and inflammation: Implications for atherosclerosis

**DOI:** 10.1016/j.metop.2026.100459

**Published:** 2026-03-15

**Authors:** Fan Fan, De-Jing Shang

**Affiliations:** a School of Life Science, Liaoning Normal University, Dalian, 116081, China; b School of Life Science, Liaoning Provincial Key Laboratory of Biotechnology and Drug Discovery, Liaoning Normal University, Dalian, 116029, China

**Keywords:** Atherosclerosis, miRNA, Macrophages, Foam cells, Lipid metabolism, Inflammation

## Abstract

Atherosclerosis (AS) is the primary pathological basis of global cardiovascular diseases. Its progression is a complex process driven by the dynamic interplay between lipid metabolism disorders and immune-inflammatory responses. Macrophages play a central role in this pathology, actively participating from early monocyte recruitment and differentiation to the formation of foam cells and the inflammation triggered by lipid accumulation. Dysfunctional macrophages are involved throughout the entire process of AS development. MicroRNAs (miRNAs) act as critical post-transcriptional regulators, modulating the intricate balance between lipid metabolism and inflammatory responses. MiRNAs contribute to lipid homeostasis by regulating key processes, including lipid uptake, cholesterol efflux, and fatty acid synthesis. Additionally, miRNAs modulate critical inflammatory signaling pathways and transcription factors, exacerbating immune responses and driving disease progression. This paper focuses specifically on macrophages, systematically reviewing the role of miRNAs as key mediators linking lipid metabolism and immune responses, and highlighting their core function and therapeutic potential in atherosclerosis.

## 1 Introduction

AS is the primary pathological cause underlying ischemic cardiovascular events. These events encompass coronary heart disease, stroke, and peripheral artery disease [1]. It remains one of the leading causes of death and disability worldwide. Despite significant advances in reducing low-density lipoprotein cholesterol (LDL-C) through treatments such as statins and proprotein convertase subtilisin/kexin type 9 (PCSK9) inhibitors, the burden of atherosclerosis-related diseases continues to rise due to increasing rates of aging and metabolic diseases.

**Fig. 1:**
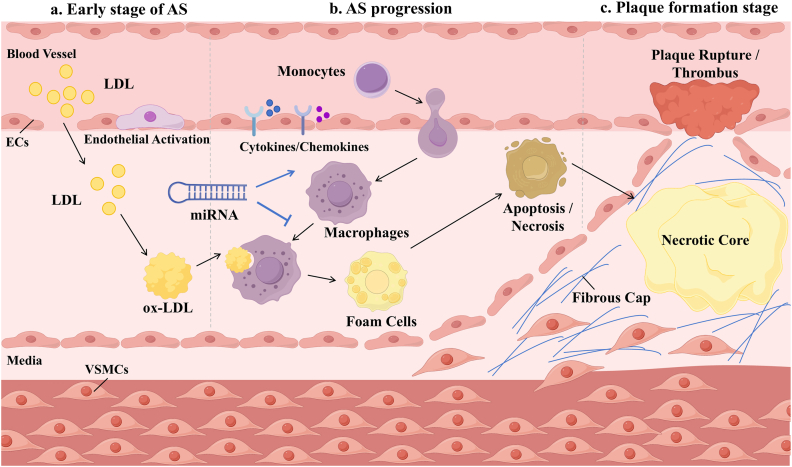
The initiation, progression, and plaque rupture process of atherosclerosis. AS develops through three main stages: (a) Early stage: Following EC damage and activation, circulating LDL penetrates the endothelium into the intima, where it is oxidatively modified into ox-LDL. (b) Progression stage: Activated ECs release cytokines and chemokines, promoting the adhesion and transmigration of circulating monocytes into the intima, which subsequently differentiate into macrophages. Macrophages engulf large amounts of ox-LDL to transform into foam cells. This cascade is intricately regulated by miRNAs, which modulate inflammation and macrophage function by targeting specific genes. As the disease progresses, a portion of macrophages and foam cells undergo apoptosis or necrosis. (c) Plaque formation stage: Apoptotic and necrotic cellular debris and lipid accumulation form a lipid-rich necrotic core in the deep intima. Meanwhile, VSMCs from the media migrate into the intima, forming a fibrous cap covering the surface of the necrotic core. Thinning of this cap causes plaque instability, triggering rupture and subsequent thrombosis. Abbreviations: AS, Atherosclerosis; ECs, Endothelial Cells; LDL, Low-Density Lipoprotein; ox-LDL, Oxidized LDL; VSMCs, Vascular Smooth Muscle Cells.

From a pathophysiological perspective, atherosclerosis is not merely a process of lipid accumulation. It is a chronic, progressive disease driven by multiple factors, including lipid disorders, endothelial dysfunction, inflammatory responses, and immune system activation (Fig. 1). Low-density lipoprotein (LDL) accumulates at sites with increased endothelial permeability and altered shear stress. In this specific microenvironment, LDL becomes susceptible to modifications such as oxidation and glycosylation. These changes result in oxidized LDL (ox-LDL) and other pro-atherogenic phenotypes. Importantly, these altered lipoproteins are more readily recognized and ingested by immune cells within the vascular wall. Risk factors such as angiotensin II, advanced glycation end-products, and smoking further exacerbate oxidative stress and endothelial damage [2]. This promotes the retention of lipoproteins in the intima, which in turn triggers local inflammation. Mononuclear cells adhere to the endothelial surface via chemokines (e.g., CCL2) and adhesion molecules (VCAM-1 and ICAM-1). They then migrate into the intima and differentiate into macrophages. These macrophages engulf modified lipoproteins, forming foam cells. The apoptosis and necrosis of foam cells lead to the expansion of the necrotic core. Meanwhile, vascular smooth muscle cells (VSMCs) migrate and synthesize a collagen matrix, forming a stabilizing fibrous cap. This process transforms simple fatty streaks into complex atheromatous plaques. The combined effects of lipid accumulation and inflammatory cell infiltration drive plaque maturation. This maturation can ultimately lead to plaque instability and rupture. Subsequent plaque rupture and thrombosis may trigger acute ischemic events, including myocardial infarction and stroke.

Macrophages are among the most abundant and functionally diverse immune cells in atherosclerotic plaques. Notably, macrophages are pivotal mediators linking lipid metabolic imbalance and inflammation in the progression of atherosclerosis. The core events of this metabolic dysregulation involve the uptake of modified LDL, disruption of cholesterol efflux pathways, and subsequent foam cell formation. In turn, lipid accumulation actively triggers inflammatory signaling. This signaling promotes cytokine secretion and, critically, further impairs cholesterol efflux, thereby perpetuating the cellular lipid burden. Macrophage metabolic dysfunction and inflammatory activation play a decisive role throughout the entire continuum of atherosclerosis, from early plaque formation to the development of advanced, complex plaques [[3], [4], [5]].

MicroRNAs (miRNAs) are endogenous small non-coding RNAs that fine-tune gene expression at the post-transcriptional level. Despite their small size, miRNAs coordinate broad regulatory programs. Analyses reveal that individual miRNAs regulate large sets of transcripts, and over 60% of human genes are under selective pressure to maintain miRNA pairing [6]. Accordingly, miRNAs influence fundamental cellular processes, including proliferation, differentiation, apoptosis, and metabolic homeostasis. Aberrant miRNA expression is a hallmark of multiple diseases and is increasingly recognized as a driver of both initiation and progression of AS through coordinated control of lipid handling and inflammatory signaling in vascular cells and macrophages [7].

## 2 MicroRNA

**Fig. 2:**
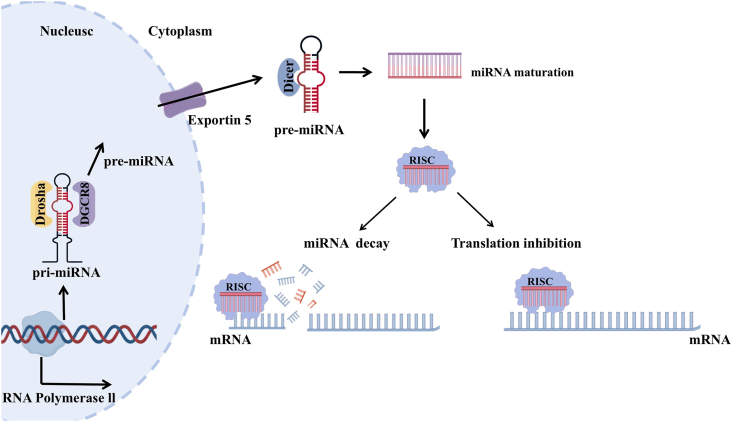
**Biogenesis of microRNAs and their two principal modes of target gene regulation.** Canonical miRNA biogenesis begins with the transcription of pri-miRNAs by RNA polymerase II, followed by nuclear processing by the Drosha/DGCR8 microprocessor complex to generate pre-miRNAs. Pre-miRNAs are exported to the cytoplasm via Exportin-5 and further processed by Dicer to produce mature miRNA duplexes, from which the guide strand is selectively incorporated into the RISC. Upon integration into the RISC, mature miRNAs modulate gene expression via two distinct modes:(i) mRNA degradation, achieved through extensive base pairing with the 3′-UTR of target transcripts; (ii) translational repression, mediated by partial complementarity that inhibits ribosome-dependent protein synthesis without immediately inducing mRNA decay.

MiRNAs are endogenous single-stranded non-coding RNAs that mediate post-transcriptional gene silencing and are generated through a tightly regulated, multi-step biogenesis process (Fig. 2). The process is initiated by RNA polymerase II transcription, which yields a primary transcript called pri-miRNA. This transcript, ranging from hundreds to thousands of nucleotides, features a characteristic stem-loop structure. This unique conformation serves as the essential substrate for subsequent processing events [8]. In the nucleus, the pri-miRNA is recognized by the microprocessor complex, composed of Drosha and its partner diGeorge syndrome critical region 8 (DGCR8). This complex executes the first crucial cleavage step, producing the precursor miRNA (pre-miRNA). The resulting pre-miRNA is approximately 70 nucleotides long and maintains a distinct hairpin structure. Subsequently, the pre-miRNA is transported from the nucleus to the cytoplasm via the nuclear export protein Exportin-5 in a Ran-GTP-dependent manner. Upon cytoplasmic entry, the RNase III enzyme Dicer processes the pre-miRNA. Dicer excises the terminal loop, generating a transient miRNA duplex of approximately 22 nucleotides. This duplex comprises the miRNA-5p and miRNA-3p strands, which display partial complementarity [9]. Crucially, the RNA-induced silencing complex (RISC) selectively incorporates the guide strand. AGO2 acts as the catalytic core and its binding assembles the functional RISC. Consequently, the complex degrades the opposing passenger strand.

Functionally, mature miRNAs operate within the RISC. Specificity is dictated by the seed sequence, comprising nucleotides 2 through 8 of the miRNA. This domain binds to the 3′-UTR of target transcripts. In mammalian cells, miRNA typically binds to its target mRNA with partial complementarity, thereby primarily inhibiting translation [10]. Fully complementary binding, however, is more likely to induce direct mRNA cleavage. This binding not only hinders protein translation but also recruits mechanisms such as deadenylation and decapping, which decrease mRNA stability and accelerate its degradation. A single miRNA species typically modulates a broad spectrum of mRNA targets. Simultaneously, the presence of binding sites for multiple distinct miRNAs within individual transcripts gives rise to a dense regulatory network. Within this architecture, a concise subset of miRNAs can orchestrate complex biological programs, including lipid metabolism and inflammatory responses.

Crucially, miRNA-mediated regulation is highly context-dependent. Expression profiles fluctuate dynamically across distinct cell lineages, developmental stages, and in response to environmental cues. Efficiency is governed by multiple variables, including transcriptional rates, RISC stoichiometry, and the structural accessibility of mRNA 3′-UTRs. These factors dictate the efficacy of miRNA biosynthesis, loading, and target engagement. Consequently, miRNAs serve as molecular bridges linking cellular states to gene expression outputs. They govern diverse physiological processes ranging from metabolic homeostasis and immune modulation to cell cycle progression and apoptosis. In multifactorial pathologies such as cardiovascular disease, this regulatory plasticity is particularly significant [11]. Analyzing miRNA signatures unveils underlying mechanisms and identifies critical control nodes. Thus, miRNAs emerge as promising candidates for diagnostic biomarkers and therapeutic intervention.

## 3 Regulation of lipid metabolism by miRNAs

### 3.1 Regulation of lipid uptake by miRNAs

The internalization of lipids by macrophages serves as the precipitating event for foam cell transformation. However, when exposed to hyperlipidemia or heightened oxidative stress, LDL infiltrating the arterial intima becomes highly susceptible to pathological modifications. These modified LDL species exhibit enhanced immunogenicity and are preferentially recognized and internalized by macrophages via specific scavenger receptors. Key scavenger receptors include Cluster of Differentiation 36 (CD36), Lectin-like oxidized low-density lipoprotein receptor-1 (LOX-1), and Scavenger Receptor Class A Member 1 (SR-A1). In contrast to the native LDL receptor (LDLR), scavenger receptors are not subject to negative feedback regulation by intracellular cholesterol levels. This lack of control permits the unrestricted uptake of ox-LDL, leading to excessive intracellular cholesterol accumulation [12]. MiRNAs govern this influx by modulating the surface density of scavenger receptors.

Multiple miRNA species have been identified that target the CD36 transcript, and by suppressing CD36 expression these miRNAs act as molecular brakes on excessive lipid accumulation [13]. One prominent regulator is miR-210-3p, which functions indirectly by directly targeting Insulin-like Growth Factor 2 (IGF2). In ox-LDL-induced macrophages, miR-210-3p overexpression suppresses IGF2/IGF2R signaling, reduces downstream NF-κB activity, and ultimately leads to a marked reduction of CD36 protein abundance, thereby mitigating lipid internalization by limiting CD36 availability [14]. In contrast, miR-758-5p targets the CD36 transcript directly. It binds the 3′-UTR and suppresses CD36 protein expression, which reduces macrophage ox-LDL internalization and lowers cellular cholesterol burden, thereby limiting foam cell formation [15]. Consistently, recent evidence from HFD *ApoE*^*−/−*^ mice shows that environmental exposure markedly downregulates miR-504-3p, a direct inhibitor of CD36; loss of miR-504-3p derepresses CD36 and drives excessive macrophage cholesterol uptake and intracellular lipid accumulation, which ultimately accelerates atherosclerotic progression [16].

Beyond CD36, other members of the scavenger receptor superfamily, including SR-A1 and SR-BII, also facilitate lipid internalization [17]. In this regulatory axis, miR-204 is transcriptionally induced by Nuclear Factor of Activated T-cells, Cytoplasmic 3 (NFATc3) and represses lipid uptake through a dual mechanism targeting both SR-A and CD36. Canonically, miR-204-5p binds the 3′-UTR of *SR-A1* mRNA and silences SR-A1 expression post-transcriptionally, while miR-204-3p shows nuclear localization and complexes with Ago2 at the CD36 promoter region, thereby impeding transcription factor recruitment and blunting CD36 transcriptional activity. Recent work further indicates that miR-204-5p also targets the 3′-UTR of *SR-BII*. By suppressing SR-BII, it may contribute to the mitigation of atherosclerotic plaque formation. Consistent with these mechanisms, in vivo validation in *ApoE*^*−/−*^ models showed that miR-204 overexpression significantly attenuates arterial lesion burden and reduces macrophage foam cell accumulation [18,19].

The LOX-1 axis is regulated by multiple miRNAs. MiR-98 directly binds the *LOX-1* 3′-UTR, suppressing LOX-1 mRNA and protein and thereby reducing macrophage ox-LDL internalization [20]. Similarly, Let-7g mimics administered to high-fat diet (HFD)-fed models mitigate arterial intimal thickening and reduce plaque formation, in parallel with marked downregulation of vascular LOX-1 [21]. In addition to receptor-specific control, miR-27a/b coordinates broader lipid-handling programs. In ox-LDL-induced macrophages, miR-27a/b overexpression reduces lipid uptake and foam-cell formation, and this is accompanied by decreased LPL expression. It also downregulates multiple lipid-entry pathways, including SR-A1, LOX-1, CD36, and CXCL16. By contrast, in HFD-fed *ApoE*^*−/−*^ mice, systemic delivery of a miR-27a/b agomir reduces aortic lesion size and lipid content [22].

### 3.2 Regulation of synthesis and esterification of cholesterol by miRNAs

Intracellular lipid homeostasis relies heavily on cholesterol esterification and lipid droplet biogenesis. These processes underpin macrophage lipid accumulation. Central to this metabolic landscape is de novo lipogenesis, which generates fatty acids and cholesterol for subsequent conversion into triglycerides and cholesteryl esters. This anabolic machinery is orchestrated by the Sterol Regulatory Element-Binding Transcription Factor 1 (SREBF1). SREBF1 acts as a master regulator, driving the expression of Fatty Acid Synthase (FAS), the rate-limiting enzyme in fatty acid synthesis. Crucially, miRNAs exert precise control over this transcriptional network. By modulating SREBF1 and FAS, they strictly dictate cellular lipid storage capacity [12,23].

One important miRNA in lipid synthesis is miR-33. miR-33a/miR-33b are intronic miRNAs within SREBF2/SREBF1 and are co-transcribed with SREBP-2 and SREBP-1, resulting in coordinated upregulation during lipogenic activation. miR-33 represses fatty acid β-oxidation by targeting CPT1A, HADHB, and CROT, which is associated with increased free fatty acids and triglyceride accumulation [24]. Parallel to fatty acid synthesis is the generation of cholesterol, a process governed by 3-hydroxy-3-methylglutaryl-CoA reductase (HMGCR). As the rate-limiting enzyme, HMGCR catalyzes the conversion of HMG-CoA to mevalonate, initiating sterol biosynthesis. This metabolic checkpoint is strictly modulated by miR-224 and miR-520d. Both microRNAs bind directly to *HMGCR* mRNA, inducing post-transcriptional silencing. Upregulation of these miRNAs correlates with a marked reduction in HMGCR protein abundance and a subsequent deceleration of cholesterol genesis [25].

Complementing de novo synthesis is the process of esterification, a critical step for lipid storage. To mitigate free cholesterol-induced membrane lipotoxicity, stressed macrophages utilize Acyl-CoA:cholesterol acyltransferase 1 (ACAT1) to esterify cholesterol, sequestering it within cytosolic lipid droplets. The miR-27a/b cluster and miR-9 act as negative regulators of cholesterol esterification in macrophages. Both directly target *ACAT1* by binding its 3′-UTR, thereby reducing ACAT1 protein abundance, limiting cholesterol ester synthesis, and ultimately attenuating foam cell formation [22,26]. The regulatory framework also involves miR-467b. In murine models, upregulation of this miRNA disrupts ACAT1 function, resulting in diminished lipid droplet biogenesis and arrested plaque progression [27].

### 3.3 Regulation of cholesterol efflux by miRNAs

To counterbalance influx and storage, macrophages rely on cholesterol efflux. Cholesterol efflux constitutes the first step of reverse cholesterol transport (RCT), through which excess sterols are conveyed from peripheral tissues to the liver and intestine for ultimate elimination. Mobilization is mediated by specific membrane transporters, principally ATP-binding cassette transporters A1 (ABCA1), ABCG1, and SR-BI. Specifically, ABCA1 transfers cholesterol to apolipoprotein A-I (ApoA-I), and ABCG1 transfers it to high-density lipoprotein (HDL). Emerging evidence indicates that miRNAs tightly govern this efflux machinery, thereby dictating the overall efficiency of RCT [28].

Specifically, miR-758 negatively regulates ABCA1, thereby impairing apoA-I-mediated cholesterol efflux. Functional assays demonstrate that miR-758 overexpression suppresses ABCA1 protein abundance. Clinical relevance is highlighted by studies in patients with obesity and metabolic syndrome. In these cohorts, plasma miR-758 levels correlate with ABCA1 expression patterns, corroborating its pivotal role in systemic cholesterol metabolism [29]. In addition to miR-758, miR-200b-3p has also been identified as a direct regulator of ABCA1. MiR-200b-3p expression is markedly upregulated in the peripheral blood of patients with AS and in ox-LDL-induced RAW264.7 macrophages. Meanwhile, ABCA1 mRNA and protein levels are significantly reduced.

Beyond direct targeting, certain miRNAs modulate ABCA1 indirectly by governing the Liver X Receptor (LXR). Upon activation in macrophages and hepatocytes, LXR functions as a master regulator of lipid homeostasis. It forms a functional heterodimer with the Retinoid X Receptor (RXR). This complex binds to specific response elements within the ABCA1 promoter, thereby driving transcriptional activation. Consequently, this pathway facilitates cholesterol transfer to ApoA-I and nascent HDL. Zhao et al. reported that in human THP-1 macrophages treated with a Peroxisome Proliferator-Activated Receptor γ (PPARγ) agonist, miR-613 binds to the 3′-UTR of *LXRα* and *ABCA1* mRNA, inhibiting their expression and thereby reducing cholesterol excretion [30]. This suggests that miR-613 negatively regulates cholesterol metabolism through two routes, directly inhibiting ABCA1 post-transcriptionally and weakening LXRα-driven ABCA1 transcription. In contrast to miR-613, miR-206 exhibits a dichotomous regulatory pattern contingent on cellular context [31]. In hepatocytes, it functions as a canonical repressor targeting the *LXRα* 3′-UTR. This interaction suppresses expression and impairs cholesterol efflux, thereby precipitating hepatic lipid accumulation and steatosis. However, in macrophages, miR-206 upregulation increases LXRα abundance. This increase potentiates the transcriptional activation of downstream targets, including ABCA1 and ABCG1, ultimately driving robust cholesterol efflux. Intriguingly, LXRα activation triggers the reciprocal repression of miR-206, establishing a negative feedback loop. Therefore, miR-206 exerts cell type–specific control over cholesterol homeostasis. Let-7c attenuates the PGC-1α/LXRα axis by targeting Peroxisome Proliferator-Activated Receptor Gamma Coactivator 1-alpha (PGC-1α), a key co-activator of LXRα. In *ApoE*^*−/−*^ models, inhibitor treatment restored ABCA1 and ABCG1 expression. This restoration limited foam cell genesis, and significantly attenuated atherosclerotic plaque burden [32].

**Fig. 3:**
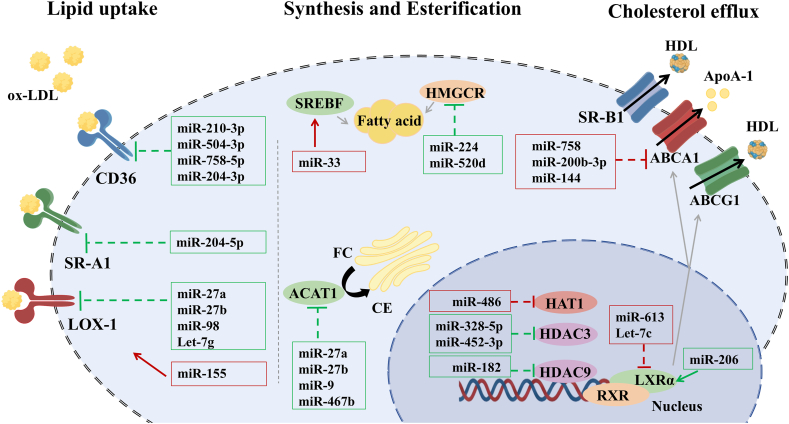
**MiRNA regulation of macrophage lipid metabolism.** MiRNA regulation of macrophage lipid uptake, synthesis, esterification, and efflux. (i) Lipid uptake: miR-210-3p, miR-204-5p, miR-27a, etc., target CD36, SR-A1, and LOX-1, respectively, attenuating lipid uptake to suppress AS; conversely, miR-155 promotes this process. (ii) Synthesis and esterification: miR-33, miR-224, etc., inhibit nuclear SREBF1 and HMGCR, while miR-27a, etc., target ACAT1 in the endoplasmic reticulum. These interactions suppress AS by mitigating lipid accumulation. (iii) Cholesterol efflux: miR-758, etc., directly target ABCA1, impairing efflux and promoting AS. In terms of nuclear regulation, miR-328-5p, etc., upregulate ABCA1 via HDAC inhibition (suppressing AS), whereas miR-613 and miR-486 downregulate ABCA1 by inhibiting LXRα and HAT1, respectively (promoting AS). Green frames indicate miRNAs that suppress AS, whereas red frames indicate miRNAs that promote AS. (For interpretation of the references to colour in this figure legend, the reader is referred to the Web version of this article.)

MiRNAs also shape the epigenetic landscape through crosstalk with histone deacetylases (HDACs) and histone acetyltransferases (HATs). Functionally, HDACs govern transcriptional accessibility. By catalyzing the removal of acetyl groups from histone tails, they facilitate chromatin compaction and gene silencing. Specific miRNAs, notably miR-328-5p and miR-452-3p, have been identified as regulators of this machinery [33,34]. MiR-328-5p directly targets the *HDAC3* 3′-UTR to restrain HDAC3 expression, thereby preserving ABCA1 levels and cholesterol efflux. The regulation of HDAC3 is further stratified by long non-coding RNAs (lncRNAs) functioning within competitive endogenous RNA (ceRNA) networks. Specifically, lncRNA Kcnq1ot1 acts as a molecular sponge for miR-452-3p.Through sequestration of this miRNA, Kcnq1ot1 releases HDAC3 from miRNA-mediated repression, leading to HDAC3 upregulation and consequent suppression of ABCA1. This Kcnq1ot1/miR-452-3p/HDAC3 axis illustrates a hierarchical signaling cascade [35]. MiR-486 functions as a post-transcriptional repressor by directly targeting the *HAT1* 3′-UTR [36]. Under physiological conditions, HAT1-mediated acetylation of lysine residues on core histone H4 promotes an open chromatin configuration and enhances transcriptional activation. HAT1 overexpression elevates *ABCA1* mRNA and protein abundance, driving enhanced cholesterol efflux. Conversely, miR-486 gain-of-function or HAT1 silencing suppresses ABCA1 expression, leading to impaired cholesterol efflux and consequent lipid accumulation (Fig. 3).

## 4 Regulation of inflammation by miRNAs

### 4.1 Regulation of macrophage polarization by miRNAs

Phenotypic polarization fundamentally dictates macrophage function within the atherosclerotic environment. Traditionally, these phenotypes are broadly categorized into classically activated (pro-inflammatory) and alternatively activated (anti-inflammatory) subsets. While classically activated macrophages prioritize pro-inflammatory responses and pathogen elimination, alternatively activated macrophages primarily govern tissue homeostasis and the resolution of inflammation [37]. Within this classical M1-M2 framework, Hildebrandt et al. used RNA sequencing to evaluate miRNA profiles in bone marrow-derived macrophages (BMDMs). These cells were stimulated with ox-LDL [38]. Their findings identify specific miRNA shifts as central drivers of atherosclerosis. Specifically, classically activated macrophages exhibited significant upregulation of pro-inflammatory transcripts compared to the alternatively activated phenotype. Upregulated species included miR-155-5p, miR-181a, miR-204-5p, miR-92a, miR-221-5p, miR-451, miR-124-3p, miR-25, and miR-127-3p. In contrast, regulatory miRNAs, such as miR-125-5p, miR-146a-3p, miR-143-3p, and miR-145-5p, showed marked downregulation. Validating these patterns in a clinical setting, Parahuleva and colleagues analyzed human advanced coronary atherosclerotic plaques. They observed elevated levels of miR-21, miR-92a, and miR-99a within the lesions. These distinct profiles suggest that specific miRNAs could serve as molecular biomarkers for plaque instability.

However, recent advancements in single-cell RNA sequencing (scRNA-seq) have refined our understanding of macrophage heterogeneity, revealing that the traditional binary paradigm is inadequate for the complex atherosclerotic microenvironment. In vivo scRNA-seq data suggest that plaque macrophages do not exist in mutually exclusive states; instead, they form a transcriptional continuum and transition through intermediate states driven by local metabolic stress. High-resolution transcriptomic profiling in models such as *ApoE*^*−/−*^ mice has delineated distinct, plaque-specific macrophage subsets. Prominent among these are tissue-resident macrophages, inflammatory macrophages, interferon-stimulated macrophages, and lipid-associated macrophages (LAMs). Notably, across the diverse spectrum of polarization states, LAMs and inflammatory macrophages remain the primary focus of profound research in metabolic inflammation. Characterized by the high expression of triggering receptor expressed on myeloid cells 2 (TREM2), major histocompatibility complex class II (MHCII), and lipid efflux-associated genes, LAMs can adapt to lipid overload and effectively counteract metabolic stress within advanced plaques. In contrast, the inflammatory subsets drive pathological responses by releasing abundant inflammatory cytokines [39,40].

Transitions between these distinct states are regulated by intricate post-transcriptional networks, in which miRNAs serve as central regulators. For instance, targeted silencing of miR-33 remodels the immune microenvironment within advanced atherosclerotic lesions [41]. ScRNA-seq analysis suggests that anti-miR-33 therapy promotes the upregulation of cholesterol efflux-related gene programs and reshapes the distribution of macrophage subpopulations. This intervention significantly reduces the proportions of MHCII^hi^ LAMs and TREM2^hi^ LAMs while preserving tissue-resident populations, ultimately promoting inflammation resolution and reducing plaque area. Furthermore, Sprenkle et al. identified the miR-23-27-24 cluster as a key regulator of LAMs, modulating both lipid uptake and inflammatory signaling [42]. Mechanistically, miR-23 directly inhibits the translational repressor *Eif4ebp2* (4E-BP2), thereby relieving the constraints on eIF4E-dependent translation. This shift in translational patterns drives macrophage polarization toward a proliferative and metabolically active phenotype.

Emerging evidence indicates that aberrant miRNA expression contributes to dysregulated inflammatory responses, a regulatory complexity that is underscored by macrophage ontogeny. These cells differentiate from monocytes, which originate from hematopoietic stem cells (HSCs). Crucially, miRNAs govern HSC self-renewal and lineage commitment. During atherogenesis, the transcription factor PU.1 promotes the differentiation of granulocyte-monocyte progenitors (GMPs) into monocyte lineages. This transcriptional program concurrently induces specific miRNAs, including miR-155, miR-146a, miR-223 and miR-21. These non-coding RNAs precisely orchestrate hematopoietic differentiation, thereby controlling macrophage maturation and fate. Notably, sustained miR-223 upregulation in hematopoietic cells is associated with a pro-inflammatory profile and accelerated atherosclerotic plaque formation [43].

### 4.2 Regulation of pro-inflammatory signaling and cytokine production by miRNAs

**NF-κB signaling pathway** The Nuclear Factor-κB (NF-κB) signaling pathway serves as a central orchestrator of immune responsiveness. Within this regulatory framework, miR-155 represents a pivotal pro-inflammatory microRNA. It specifically modulates NF-κB signaling, thereby dictating macrophage functionality and the intensity of the inflammatory response. Pathogen challenge rapidly induces miR-155 transcription in macrophages [44]. This induction relies on key transcription factors, specifically NF-κB and activator protein 1 (AP-1). These factors bind directly to the promoter region of the MIR155 host gene (MIR155HG). Crucially, the identification of a functional NF-κB p65-responsive element within this promoter confirms NF-κB as a direct transcriptional activator. Upon induction, miR-155 establishes a positive feedback loop. Validated targets include TAK1-binding protein 2 (TAB2), inhibitor of nuclear factor-κB kinase subunit ε (IKKε), and NF-κB-inducing kinase (NIK) [45]. By disrupting these inhibitory mechanisms, miR-155 sustains NF-κB signaling amplitude, thereby driving prolonged secretion of pro-inflammatory cytokines. Counteracting the pro-inflammatory drivers, miR-146a and miR-146b function as critical negative feedback regulators of innate immunity [46]. These miRNAs attenuate NF-κB signaling by targeting key upstream adaptors: interleukin-1 receptor-associated kinase 1 (IRAK1) and TNF receptor-associated factor 6 (TRAF6). Exogenous stimulation of macrophages activates NF-κB, which in turn induces the expression of miR-146. The mature miR-146 transcripts then bind to the 3′-UTRs of *IRAK1* and *TRAF6* mRNAs, resulting in the repression of translation and a subsequent reduction in the abundance of these proteins. By disrupting MyD88-mediated signal transduction, this blockade forms a negative feedback loop that restrains NF-κB hyperactivation. Another critical regulatory node involves miR-148a, which functions by directly targeting IKKβ [47]. As a core catalytic subunit of the signaling cascade, IKKβ activity promotes the nuclear translocation of the p65 complex. Elevating miR-148a levels suppresses IKKβ protein expression. Consequently, this inhibition constrains NF-κB activity and mitigates the inflammatory burden within atherosclerotic plaques.

A subset of miRNAs are critical modulators of inflammation, primarily by targeting core components of the Toll-like receptors 4 (TLR4)/NF-κB signaling axis. For instance, the miR-200b/c cluster targets the 3′-UTR of *TLR4* [48]. By dampening this innate immune response, these miRNAs mitigate inflammation and associated tissue injury. Parallel regulatory effects are observed with miR-204-5p [49]. These findings highlight the therapeutic potential of miR-200b/c and miR-204-5p in modulating atherosclerotic lipid metabolism. Both miR-515-5p and miR-16 have been identified as TLR4 repressors [50,51]. Evidence from chondrocyte models demonstrates that miR-515-5p upregulation reduces apoptosis and inflammatory signaling, conferring cellular protection. Likewise, in intestinal epithelial cells, miR-16 inhibits lipopolysaccharide (LPS)-induced NF-κB activation via TLR4 downregulation. This suppression effectively limits epithelial damage.

**MAPK signaling pathway** The Mitogen-Activated Protein Kinase (MAPK) signaling cascade represents a fundamental transduction mechanism. Within the pathogenesis of atherosclerosis, MAPK signaling critically activates immune effectors and promotes the release of pro-inflammatory mediators. Studies have shown that miR-532-3p exerts anti-inflammatory effects in macrophages by targeting the ASK1-p38 MAPK signaling axis [52]. Apoptotic signal-regulating kinase 1 (ASK1) is an upstream kinase in the MAPK pathway that participates in activating p38 MAPK and JNK, especially under oxidative stress or inflammatory stimulation. Experiments in the human THP-1 macrophage model have shown that transfection with miR-532-3p mimics significantly reduces the mRNA and protein expression of ASK1, inhibiting the phosphorylation and nuclear translocation of p38 MAPK. This results in a reduction in the release of pro-inflammatory cytokines, including Tumor necrosis factor α (TNF-α), IL-6, and IL-2. Consequently, miR-532-3p is considered a negative regulator, and its upregulation helps attenuate the pro-inflammatory phenotype of macrophages, exhibiting potential anti-inflammatory effects. In contrast, miR-101 acts as a pro-inflammatory driver within the MAPK network [53]. Mechanistically, it targets the 3′-UTR of mitogen-activated protein kinase phosphatase 1 (MKP-1). MKP-1 serves as a critical negative regulator by dephosphorylating and inactivating key kinases, particularly p38 and JNK. Upregulation of miR-101 suppresses MKP-1 abundance. By prolonging p38 and JNK phosphorylation, the loss of phosphatase activity sustains MAPK signaling and enhances the release of inflammatory mediators, including TNF-α and IL-6. Ultimately, miR-101 exacerbates pathology by compromising the intrinsic feedback inhibition of the MAPK cascade.

**JAK-STAT3 signaling pathway** The Janus kinase/Signal transducer and activator of transcription 3 (JAK/STAT3) pathway constitutes a fundamental signaling conduit. Within the atherosclerotic milieu, JAK1 serves as a pivotal tyrosine kinase subject to regulation by specific microRNAs. Specifically, miR-22-3p directly binds to the JAK1 transcript, suppressing its expression and thereby blocking downstream JAK/STAT3 signaling activation, which attenuates JAK1-driven pro-inflammatory responses [54]. Analogous to miR-22-3p, miR-107 regulates the JAK/STAT3 axis by targeting JAK1 [55]. In cell models mimicking atherosclerotic conditions, miR-107 overexpression significantly reduces JAK1 abundance and STAT3 phosphorylation. This molecular blockade translates to inhibited cell proliferation and migration. MiR-155 targets *SOCS1* mRNA to modulate this pathway [56]. Suppressor of SOCS1 acts as a critical checkpoint within the JAK/STAT signaling architecture. It primarily restricts Signal Transducer and Activator of Transcription 1 (STAT1) and STAT3 activation by inhibiting JAK1 kinase activity. Stimulation of macrophages with LPS and interferon-γ (IFN-γ) upregulates miR-155. The resulting reduction in SOCS1 protein alleviates the suppression of JAK1. In the absence of regulatory restraint, JAK1-dependent STAT1 phosphorylation enhances the transcription of pro-inflammatory mediators, including TNF-α, IL-6, and iNOS. Collectively, miR-22-3p, miR-107, and miR-155 orchestrate atherogenesis by modulating the JAK-STAT signaling architecture. By governing macrophage polarization, cellular proliferation, and migration, this regulatory axis highlights the central role of the JAK-STAT pathway in disease progression.

**Wnt/β-catenin signaling pathway** The Wntβ-catenin signaling pathway constitutes a pivotal driver of atherogenesis. Activation of this axis governs critical pathological processes, including inflammatory responses and plaque stability. MiR-494-3p modulates macrophage polarization by repressing Wnt signaling [57]. Mechanistically, inhibition of miR-494-3p restores Wntβ-catenin signaling by targeting LRP6 and transducin β-like 1 X-linked (TBL1X). This signaling recovery promotes a transition toward the alternatively activated macrophage state. Accordingly, the expression of CD206, IL-10, TREM2, and CD163 is significantly upregulated. In vivo, this intervention effectively reduces macrophage content and enhances plaque stability in HFD mice. The regulation of miR-342-5p follows similar patterns. In the arterial tissue of *ApoE*^*−/−*^ mice, this microRNA is significantly upregulated. Concurrently, levels of Wnt3a and the downstream effector β-catenin, are markedly suppressed. These inverse correlations suggest that miR-342-5p actively represses the Wnt3a/β-catenin axis during atherogenesis [58]. Originating primarily from macrophages, elevated miR-342-5p expression closely tracks with the classically activated phenotype. Furthermore, inhibiting miR-342-5p reduces plaque complexity by decreasing macrophage infiltration and neovascularization. This intervention also attenuates arterial lipid deposition and lowers plasma levels of TNF-α and IFN-γ. Beyond its previously described functions, miR-342-5p further expands its regulatory scope by targeting AKT1. By inhibiting this kinase, it synergizes with miR-155 to reinforce the pro-inflammatory state, thereby fueling atherosclerosis.

**PI3K/AKT signaling pathway** The phosphoinositide 3-kinase (PI3K)/protein kinase B (AKT) pathway represents another central signaling hub. In the context of macrophage biology, PI3K/AKT signaling drives polarization toward the alternatively activated phenotype. Exemplifying this protective mechanism, miR-223 exerts anti-atherogenic effects by robustly activating PI3K/AKT signaling [59]. In the context of LPS-induced inflammatory stimulation, elevated miR-223 levels potentiate AKT phosphorylation, which in turn dampens TLR4/NF-κB signaling and restrains the inflammatory response. Beyond inflammation control, this pathway also regulates lipid handling. miR-223 promotes the expression of ABCA1 [60]. Ultimately, by activating the PI3K/AKT pathway, miR-223 coordinates a dual defense strategy. It suppresses inflammation and enhances reverse cholesterol transport, effectively slowing plaque initiation and progression. Conversely, miR-29a confers protection against atherosclerosis by suppressing the PI3K/AKT/mTOR signaling axis. Mechanistically, miR-29a targets the mRNA of the PI3K catalytic subunit α (PIK3CA) [61]. This interaction suppresses protein expression and PI3K signaling, thereby diminishing AKT and mTOR phosphorylation. mTOR functions as a negative regulator of autophagy, and its suppression is a prerequisite for the induction of the autophagic pathway. Overexpression of miR-29 leads to the upregulation of key autophagy markers, including Beclin autophagy-related 1 (Beclin-1) and microtubule-associated protein 1A/1B-light chain 3-II (LC3-II). Concurrently, macrophages shift toward the alternatively activated phenotype. This is defined by elevated levels of Arg-1, mannose receptor (Mrc-1), and IL-10. In contrast, markers of classical activation, such as iNOS, IL-1β, and IFN-γ, decline. Ultimately, miR-29a overexpression significantly reduces macrophage accumulation and limits overall plaque burden.

**Fig. 4:**
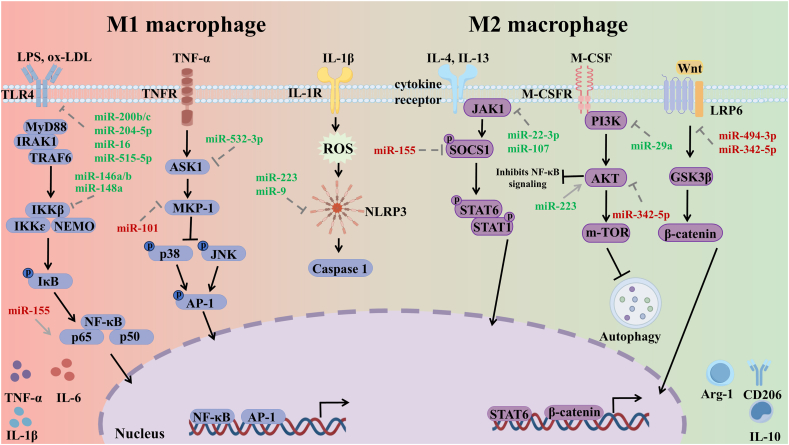
**MiRNA regulation of macrophage inflammatory signaling pathways.** MiRNAs regulate signaling pathways to govern macrophage polarization toward pro-inflammatory M1 or anti-inflammatory M2 phenotypes. (i) M1 Polarization (Left). Driven by TLR4/NF-κB, MAPK, and NLRP3 pathways. Anti-inflammatory miRNAs (Green): miR-200b/c, miR-204-5p, miR-16, miR-515-5p, miR-146a/b, miR-148a, miR-532-3p, miR-223, and miR-9 suppress inflammatory signaling. Pro-inflammatory miRNAs (Red): miR-155 and miR-101 promote pathway activation. M1 polarization is characterized by the secretion of IL-6, TNF-α, and IL-1β. (ii) M2 Polarization (Right). Mediated by JAK/STAT, PI3K/AKT, and Wnt/β-catenin pathways. M2-promoting miRNAs (Green): miR-22-3p, miR-107, miR-29a, and miR-223 facilitate polarization; miR-223 activates AKT to inhibit NF-κB signaling. M2-inhibiting miRNAs (Red): miR-494-3p and miR-342-5p repress M2 signaling. M2 polarization is characterized by the secretion of Arg-1, CD206, and IL-10. (For interpretation of the references to colour in this figure legend, the reader is referred to the Web version of this article.)

**NLRP3** The NLRP3 serves as a crucial intracellular pattern recognition receptor (PRR) of the NOD-like receptor (NLR) family. Activation of the NLRP3 inflammasome in macrophages directly promotes cellular apoptosis within the plaque, contributing to the inflammatory pathology of atherosclerosis. MiR-223 serves as a pivotal negative regulator of NLRP3 inflammasome activation in macrophages [62]. Mechanistically, miR-223 binds to the 3′-UTR of *NLRP3* mRNA. This interaction represses translation, thereby reducing NLRP3 expression and preventing inflammasome assembly. Elevated miR-223 levels curb the secretion of key inflammatory products, notably IL-1β and IL-18. This action attenuates the overall inflammatory cascade and limits macrophage pyroptosis. Conversely, reduced miR-223 expression in atherosclerotic lesions leads to NLRP3 hyperactivation. This exacerbates macrophage inflammation and accelerates plaque progression and instability. In vivo evidence from *ApoE*^*−/−*^ mice corroborates this. Administration of miR-223 agomir significantly reduced arterial plaque formation [63]. Counteracting pro-atherogenic signals, miR-9 functions as a negative regulator of NLRP3 inflammasome activation in macrophages [64]. In vitro evidence from oxidized ox-LDL-induced THP-1 macrophages confirms this. Upregulation of miR-9 significantly suppresses NLRP3 protein expression and consequent inflammasome activation. This suppression results in attenuated pro-caspase-1 cleavage. Accordingly, the maturation and secretion of IL-1β and IL-18 diminish. Conversely, silencing miR-9 enhances NLRP3 expression and amplifies inflammatory factor release. This imbalance drives local inflammation (Fig. 4).

## 5 miRNAs regulation of lipid-inflammation crosstalk

### 5.1 Lipid accumulation induces inflammatory reactions

Metabolic disorders compromise adipose tissue function. This dysfunction alters circulating miRNA profiles. MiRNAs serve as more than passive biomarkers; they actively drive inflammation. They modulate this process by perturbing specific lipid metabolism pathways. Within macrophages, aberrant miRNAs regulate inflammatory cytokines and chemokines. This control primarily occurs through post-transcriptional mechanisms. Such regulatory events can either exacerbate or resolve the inflammatory response. MiRNAs thus emerge as central mediators linking lipid dysregulation to inflammation [65,66].

MiR-34a exemplifies this critical regulatory mechanism. It targets Sirtuin 1 (SIRT1), an NAD^+^-dependent deacetylase, via post-transcriptional inhibition. Under homeostatic conditions, SIRT1 activates the nuclear receptor LXRα [67]. Oxidative stress triggers aberrant miR-34a upregulation, thereby inhibiting SIRT1 translation. Consequently, SIRT1 deficiency renders LXRα transcriptionally inactive. This inactivation prevents ABCA1 and ABCG1 induction and blocks cholesterol efflux. Simultaneously, impaired deacetylation sustains the transcription factor NF-κB in an active state. Prolonged NF-κB activation drives the release of cytokines like IL-6 and TNF-α. Furthermore, intracellular lipid accumulation promotes the formation of cholesterol crystals. These crystals activate the NLRP3 inflammasome, triggering IL-1β maturation. Ultimately, miR-34a fosters a feed-forward loop linking lipid overload to immune activation [68].

MiR-144 destabilizes plaques by compromising RCT. MiR-144 binds to the 3′-UTR of *ABCA1* mRNA. This interaction represses ABCA1 expression and hinders cholesterol efflux to ApoA-I. Impaired efflux causes lipid retention, a primary driver of foam cell formation. Chronic lipid overload induces oxidative stress through ROS accumulation. Beyond lipid metabolism, miR-144 impairs the nuclear factor erythroid 2-related factor 2 (Nrf2) signaling pathway. This suppression weakens antioxidant defenses and aggravates redox imbalances. Consequently, the oxidative environment triggers NF-κB activation. Activated NF-κB boosts the secretion of inflammatory cytokines like TNF-α and IL-6. Essentially, miR-144 fuels a vicious cycle connecting lipid toxicity with inflammation. This synergy accelerates the progression of atherosclerotic plaques [69,70].

### 5.2 Inflammation-induced disturbances in lipid metabolism

MiRNA expression exhibits rapid plasticity in response to environmental and inflammatory stimuli. Physiologically, inflammation induces specific miRNAs to restrict lipid retention and avert lipotoxicity. Conversely, aberrant miRNA signaling compromises lipid transport and facilitates the deposition of atherogenic lipids, such as ox-LDL. This maladaptation establishes a pathogenic positive feedback loop that perpetuates metabolic dysfunction [71]. Thus, miRNAs act as dynamic integrators of metabolic status and immune activation.

Notably, HFD feeding in mice upregulates miR-802 in both adipose tissue and the liver. MiR-802 targets the transcript of TRAF3. Reduced TRAF3 levels relieve the inhibition on NF-κB signaling, thereby driving its constitutive activation. Once active, NF-κB induces chemokines and the lipid regulator SREBP1. These chemokines recruit circulating monocytes to the adipose compartment. Infiltrating cells adopt a pro-inflammatory phenotype and sustain the local inflammatory microenvironment. Concurrently, SREBP1 and NF-κB signaling intensifies de novo lipogenesis. Through TRAF3, miR-802 synchronizes inflammatory signaling with lipid synthesis. It acts as a pivotal nexus between metabolic and immune dysfunction [72,73].

MiR-182 illustrates the complex interplay between lipid metabolism and immune homeostasis. In vitro studies using RAW264.7 macrophages reveal an anti-inflammatory function. Specifically, miR-182-5p mimics downregulate TLR4 expression following LPS stimulation. This suppression reduces TNF-α and IL-6 secretion while curbing lipid accumulation [74]. In contrast, in vivo models uncover a conflicting, pro-atherogenic role. In *ApoE*^*−/−*^ mice, miR-182 inhibits HDAC9 to upregulate lipoprotein lipase (LPL) [75]. This mechanism drives systemic hyperlipidemia and elevates pro-inflammatory cytokines. Thus, miR-182 exhibits a context-dependent duality. It appears protective in hepatic circulation but promotes inflammation within plaques. Such opposing effects underscore how miR-182 integrates lipid handling with immune signaling. This crosstalk fundamentally influences metabolic disease progression.

### 5.3 Regulation of lipophagy by miRNAs

Lipophagy, defined as lipid droplet-selective autophagy, links lipid metabolism with inflammatory responses. It operates specifically within the macrophage and the plaque microenvironment. Triggered by lipid overload, this process degrades accumulated lipid droplets. It also clears damaged organelles and misfolded proteins. Beyond waste clearance, the autophagic machinery preserves immune function. It participates in antigen processing and modulates inflammatory signaling. Furthermore, this mechanism governs cell fate decisions like apoptosis and necrosis. It serves as a primary adaptation to cellular stress. Consequently, robust autophagic flux is essential for inhibiting chronic inflammation. It maintains homeostasis within the macrophage niche [76].

MiR-33 acts as a convergence point for autophagy and lipid metabolism [77]. MiR-33 targets the autophagy genes Autophagy-related 5 (ATG5) and Beclin-1. ATG5 facilitates autophagosome extension and closure. Beclin-1 initiates nucleation by activating the Class III PI3K complex. Overexpressing miR-33 suppresses these proteins and blunts autophagic flux. Consequently, blocked lipophagy causes lipid droplet and cholesterol buildup. Stalled clearance mechanisms elevate oxidative stress, which triggers cytokines like TNF-α and IL-6 to exacerbate the inflammatory cascade. Similarly, miR-30a impairs lipophagy by targeting core autophagy regulators ATG5 and Beclin-1. Elevated miR-30a represses ATG5 and Beclin-1 protein abundance, blunting autophagic flux and weakening lysosome-directed lipid droplet turnover. Consequently, defective lipophagy drives lipid droplet persistence and cholesterol accumulation. The ensuing lipotoxic burden enhances ROS production and amplifies pro-inflammatory outputs, including cytokines such as TNF-α and IL-6, thereby reinforcing the metabolic inflammatory cascade [78].

Wang et al. demonstrated that overexpressing miR-761 enhances autophagic activity in ox-LDL-induced macrophages [79]. This induction upregulates Beclin-1 while reducing Sequestosome 1 (p62) levels. Together, these changes indicate a robust boost in autophagic flux, which subsequently depletes intracellular lipid droplet stores. Furthermore, it curbs the secretion of cytokines like IL-1β and IL-18. MiR-761 targets and represses mTOR, thereby relieving the inhibition on the pivotal autophagy initiator Unc-51 Like Autophagy Activating Kinase 1 (ULK1). The miR-761/mTOR/ULK1 axis promotes efficient lipid processing while limiting inflammation.

**Fig. 5:**
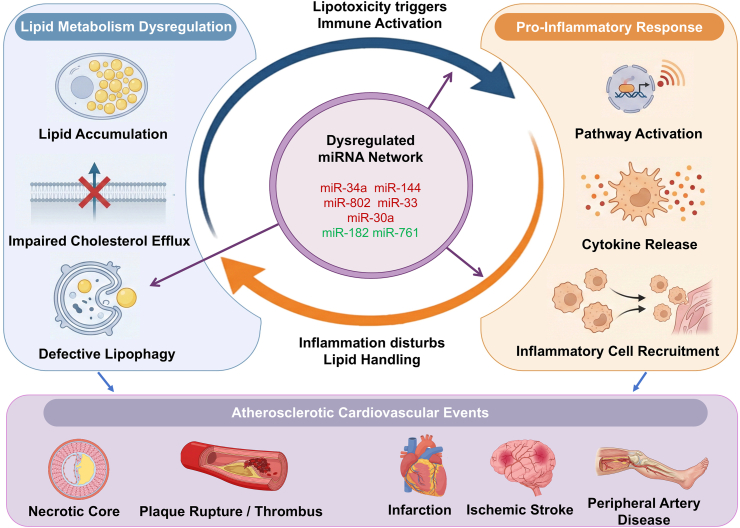
**A dysregulated miRNA network mediates the reciprocal crosstalk between lipid metabolism and inflammation.** (i) Lipid-driven inflammation (Blue arrows): Lipid accumulation, impaired cholesterol efflux, and defective lipophagy induce alterations in miRNA expression, thereby translating metabolic stress into immune activation. (ii) Inflammation exacerbates metabolic dysregulation (Orange arrows): The inflammatory microenvironment defined by pathway activation, cytokine release, and cell recruitment reshapes the miRNA profile, thereby promoting de novo lipogenesis and lipid retention. (Center) Regulatory network: Red-labeled miRNAs drive the persistence of metabolic inflammation by promoting defective lipophagy and impairing cholesterol efflux (miR-34a, miR-144, miR-802, miR-33, miR-30a), whereas green-labeled miRNAs exert protective regulatory functions aimed at restoring metabolic and immune homeostasis (miR-182, miR-761). (iii) Atherosclerotic Cardiovascular Events: The persistent feedback loop between lipotoxicity and inflammation exacerbates plaque vulnerability, driving necrotic core expansion and predisposing the lesion to rupture or thrombosis. These pathological processes ultimately result in clinical outcomes such as myocardial infarction, ischemic stroke, and peripheral artery disease. (For interpretation of the references to colour in this figure legend, the reader is referred to the Web version of this article.)

In summary, a continuous positive feedback loop between lipotoxicity and inflammation drives plaque instability, a process fueled by pathogenic miRNAs (Fig. 5). By simultaneously stalling lipophagy and impairing cholesterol efflux, these miRNAs promote the irreversible accumulation of subintimal foam cells, ultimately forcing lipid-overloaded macrophages into apoptosis. The subsequent defect in efferocytosis leaves apoptotic bodies uncleared, allowing them to progress to secondary necrosis and directly expand the necrotic core. Parallel inflammatory pathways degrade the protective fibrous cap, destroying plaque integrity and precipitating atherothrombosis. Clinically, this cascade manifests as catastrophic cardiovascular events, including acute myocardial infarction, ischemic stroke, or peripheral artery disease [80]. Therefore, therapeutically targeting specific miRNAs presents a highly promising strategy to disrupt this deleterious lipid-inflammatory axis, stabilize vulnerable lesions, and prevent these severe clinical endpoints [81]. Table 1 summarizes the key miRNAs and their targets that regulate macrophage functions in atherosclerosis.

**Table 1:** MicroRNAs regulating macrophage lipid metabolism and polarization in atherogenesis.

MicroRNA	Regulated Process	Targets	Functional Outcome	Impact on Atherogenesis	Ref.
miR-210-3p	Lipid uptake	IGF2	Inhibits lipid uptake and foam cell formation	Anti-atherogenic	[14]
miR-758-5p	Lipid uptake	CD36	Inhibits lipid uptake and foam cell formation	Anti-atherogenic	[15]
miR-504-3p	Lipid uptake	CD36	Inhibits lipid uptake and foam cell formation	Anti-atherogenic	[16]
miR-204-5p	Lipid uptake/M2↑	SR-A1/SR-BII/TLR4	Inhibits lipid uptake and foam cell formation/Inhibits TLR4/NF-κB signaling, leading to reduced pro-inflammatory cytokine production	Anti-atherogenic	[18,49]
miR-98	Lipid uptake	LOX-1	Inhibits lipid uptake and foam cell formation	Anti-atherogenic	[20]
Let-7g	Lipid uptake	LOX-1	Inhibits lipid uptake and foam cell formation	Anti-atherogenic	[21]
miR-33	Cholesterol synthesis/M1↑	ATG5/Beclin-1	Promotes lipid synthesis/Inhibits lipophagy, promoting sustained inflammatory signaling	Atherogenic	[24,77]
miR-224	Cholesterol synthesis	HMGCR	Inhibits cholesterol synthesis	Anti-atherogenic	[25]
miR-520d	Cholesterol synthesis	HMGCR	Inhibits cholesterol synthesis	Anti-atherogenic	[25]
miR-27a/b	Cholesterol esterification	ACAT1	Inhibits cholesterol esterification and foam cell formation	Anti-atherogenic	[22]
miR-9	Cholesterol esterification/M2↑	ACAT1/NLRP3	Inhibits cholesterol esterification/Inhibits the NLRP3 inflammasome	Anti-atherogenic	[26,64]
miR-467b	Cholesterol esterification	ACAT1	Inhibits cholesterol esterification and foam cell formation	Anti-atherogenic	[27]
miR-758	Lipid efflux	ABCA1	Inhibits cholesterol efflux	Atherogenic	[29]
miR-613	Lipid efflux	LXRα/ABCA1	Inhibits cholesterol efflux	Atherogenic	[30]
miR-206	Lipid efflux	/	Promotes cholesterol efflux	Anti-atherogenic	[31]
Let-7c	Lipid efflux	PGC-1α	Inhibits cholesterol efflux	Atherogenic	[32]
miR-328-5p	Lipid efflux	HDAC3	Upregulates ABCA1 expression, promoting cholesterol efflux	Anti-atherogenic	[33]
miR-452-3p	Lipid efflux	HDAC3	Upregulates ABCA1 expression, promoting cholesterol efflux	Anti-atherogenic	[35]
miR-486	Lipid efflux	HAT1	Downregulates ABCA1 expression, inhibiting cholesterol efflux	Atherogenic	[36]
miR-200b	M2↑	TLR4	Inhibits TLR4/NF-κB signaling, leading to reduced pro-inflammatory cytokine production	Anti-atherogenic	[48]
miR-200c	M2↑	TLR4	Inhibits TLR4/NF-κB signaling, leading to reduced pro-inflammatory cytokine production	Anti-atherogenic	[48]
miR-515-5p	M2↑	TLR4	Inhibits TLR4/NF-κB signaling, leading to reduced pro-inflammatory cytokine production	Anti-atherogenic	[50]
miR-16	M2↑	TLR4	Inhibits TLR4/NF-κB signaling, leading to reduced pro-inflammatory cytokine production	Anti-atherogenic	[51]
miR-146a	M2↑	IRAK1/TRAF6	Suppresses the NF-κB signaling pathway	Anti-atherogenic	[46]
miR-146b	M2↑	IRAK1/TRAF6	Suppresses the NF-κB signaling pathway	Anti-atherogenic	[46]
miR-148a	M2↑	IKKβ	Suppresses the NF-κB signaling pathway	Anti-atherogenic	[47]
miR-155	M1↑	SOCS1/SHIP1	Activates the NF-κB signaling pathway	Atherogenic	[44,56]
miR-532-3p	M2↑	ASK1	Suppresses the MAPK signaling pathway	Anti-atherogenic	[52]
miR-101	M1↑	MKP-1	Activates the MAPK signaling pathway	Atherogenic	[53]
miR-223	M2↑	NLRP3	Inhibits the NLRP3 inflammasome	Anti-atherogenic	[62,63]
miR-22-3p	M2↑	JAK1	Suppresses the JAK/STAT3 signaling pathway	Anti-atherogenic	[54]
miR-107	M2↑	JAK1	Suppresses the JAK/STAT3 signaling pathway	Anti-atherogenic	[55]
miR-29a	M2↑	PIK3CA	Suppresses the PI3K/AKT/mTOR signaling pathway, leading to autophagy activation	Anti-atherogenic	[61]
miR-494-3p	M1↑	LRP6/TBL1X	Suppresses the Wnt/β-catenin signaling pathway	Atherogenic	[57]
miR-342-5p	M1↑	Wnt3a	Suppresses the Wnt/β-catenin signaling pathway	Atherogenic	[58]
miR-34a	M1↑	SIRT1	Activates the NF-κB signaling pathway/Inhibits cholesterol efflux	Atherogenic	[67,68]
miR-144	M1↑	Nrf2/ABCA1	Activates the NF-κB signaling pathway/Inhibits cholesterol efflux	Atherogenic	[69,70]
miR-802	M1↑	TRAF3	Activates the NF-κB signaling pathway/Promotes lipid synthesis	Atherogenic	[72,73]
miR-182	M2↑	TLR4	Inhibits TLR4/NF-κB signaling, leading to reduced pro-inflammatory cytokine production	Anti-atherogenic	[74]
miR-30a	M1↑	ATG5/Beclin-1	Inhibits lipophagy, promoting sustained inflammatory signaling	Atherogenic	[78]
miR-761	M2↑	mTOR	Activates lipophagy, inhibiting inflammatory signaling	Anti-atherogenic	[79]

## 6 Harnessing MicroRNAs for diagnostics and therapy

Circulating miRNAs have emerged as promising non-invasive biomarkers with significant diagnostic and prognostic utility in cardiovascular medicine. Utilizing multi-miRNA panels enhances diagnostic sensitivity, successfully differentiating CAD patients from healthy individuals [82]. Specifically, studies have shown that plasma microvesicular miR-126-5p and miR-223-3p correlate with TIMI flow scores in acute coronary syndrome (ACS), yielding exceptional Area Under the Curve (AUC) values of 0.918 and 1.00, respectively [83]. Beyond these diagnostic metrics, miRNAs have emerged as powerful tools for cardiovascular liquid biopsy, offering distinct advantages over traditional imaging such as minimal invasiveness and high reproducibility. Crucially, they detect early, subclinical lesions often missed by conventional methods. Furthermore, technological refinements like digital PCR enable the precise quantification of low-abundance targets, facilitating longitudinal monitoring. Thus, miRNAs serve as non-invasive biomarkers poised for integration into standardized atherosclerosis risk protocols [84].

MiRNAs orchestrate lipid metabolism and immune responses by modulating gene networks, making them promising targets for therapeutic intervention [85]. Current development focuses on two primary strategies. First, miRNA mimics restore the levels of protective miRNAs. Conversely, anti-miRNA oligonucleotides selectively inhibit pathogenic variants. Recent advances have optimized diverse miRNA delivery platforms. Key categories include non-viral nanoparticles, such as liposomes and polymers, alongside bio-inspired extracellular vesicles (EVs). Non-viral systems offer distinct advantages over traditional viral vectors, specifically exhibiting superior biocompatibility and reduced immunogenicity. These systems allow for robust manufacturing scalability. As a result, non-viral vectors have become the preferred strategy for therapeutic development [86].

## 7 Summary and outlook

Atherosclerosis is a chronic pathology fundamentally driven by the convergence of macrophage lipid dysregulation and persistent inflammation. This review highlights the pivotal regulatory role of miRNAs within this complex landscape. In the domain of lipid metabolism, miRNAs strictly govern intracellular lipid fluxes by modulating the expression of scavenger receptors responsible for internalization, regulating the enzymatic machinery of cholesterol biosynthesis and esterification, and controlling the transporters essential for reverse cholesterol transport. Concurrently, in the context of inflammation, these non-coding RNAs dictate macrophage phenotypic plasticity by regulating key signaling cascades, including the NF-κB and JAK-STAT pathways. They also modulate NLRP3 inflammasome assembly, thereby determining the balance between pro-inflammatory activation and immune resolution. Crucially, miRNAs act as central integrators of the lipid-inflammation crosstalk. They orchestrate feedback loops where metabolic stress triggers immune activation and inflammatory signals disrupt lipid handling, while also regulating lipophagy to preserve cellular homeostasis.

The clinical relevance of targeting these miRNA-driven networks extends far beyond maintaining cellular homeostasis. The unchecked progression of macrophage lipid accumulation and sustained inflammatory responses directly precipitates plaque vulnerability and rupture. This critical pathophysiological transition bridges the localized molecular cross-talk to devastating systemic consequences, primarily clinically relevant atherosclerotic cardiovascular events such as acute myocardial infarction, ischemic stroke, and peripheral artery disease. Consequently, translating miRNA-based interventions into clinical practice provides a targeted therapeutic strategy to mitigate residual cardiovascular risk, thereby improving clinical outcomes for patients with advanced atherosclerosis.

Despite the promising therapeutic potential of miRNAs, their translation from bench to bedside is currently hindered by multifaceted biological and technical limitations. The inherent pleiotropy of miRNAs remains a primary hurdle for their therapeutic use. Since a single miRNA regulates multiple targets across diverse pathways, this broad activity complicates precision drug development. While current vectors have improved stability, efficient delivery remains a critical challenge [87]. The most significant issues involve overcoming suboptimal biodistribution and technical barriers such as endosomal escape and RISC loading. Notably, current vectors accumulate preferentially in clearance organs like the liver and spleen, while achieving insufficient concentrations in arterial plaques. Beyond these technical hurdles, translating findings from animal models to clinical settings presents further difficulties. A major limitation is that animal models often fail to recapitulate the complex pathology of human plaques, a biological discrepancy that impedes successful clinical translation.

To advance the field, future studies must elucidate miRNA functions across the heterogeneous plaque microenvironment, encompassing immune, vascular smooth muscle, and endothelial compartments. Furthermore, integrating multi-omics approaches will deepen regulatory understanding. Synergizing transcriptomics, proteomics, and single-cell data uncovers complex networks. Clinical success also hinges on optimizing miRNA delivery systems. Development must prioritize platforms with high selectivity and efficacy. With these advancements, miRNAs are poised to become powerful tools for treatiing atherosclerosis and revolutionizing precision cardiovascular medicine.

**Table:** Abbreviations

AS	Atherosclerosis
miRNAs	MicroRNAs
LDL-C	low-density lipoprotein cholesterol
PCSK9	proprotein convertase subtilisin/kexin type 9
LDL	Low-density lipoprotein
ox-LDL	oxidized LDL
VSMCs	vascular smooth muscle cells
ac-LDL	acetylated LDL
LOX-1	low-density lipoprotein receptor-1
SR-A1	Scavenger Receptor Class A Member 1
LDLR	LDL receptor
ACAT1	Acyl-CoA:cholesterol acyltransferase 1
ApoA-I	apolipoprotein A-I
ABCA1	ATP-binding cassette transporters A1
HDL	high-density lipoprotein
NF-κB	Nuclear Factor-κB
STAT1	Signal Transducer and Activator of Transcription 1
TLR	Toll-like receptors
MAPK	Mitogen-Activated Protein Kinase
MCP-1	Monocyte Chemoattractant Protein-1
JAK/STAT	Janus kinase/Signal transducer and activator of transcription
TNF-α	Tumor necrosis factor α
IL-1β	Interleukin-1β
IL-6	Interleukin-6
NLRP3	NOD-like receptor family pyrin domain containing 3
3′-UTR	3′-untranslated region
DGCR8	diGeorge syndrome critical region 8
AGO	Argonaute
pre-miRNA	precursor miRNA
RISC	RNA-induced silencing complex
FOXO1	Forkhead box O1
NFATc3	Nuclear Factor of Activated T-cells, Cytoplasmic 3
HFD	high-fat diet
SREBF1	Sterol Regulatory Element-Binding Transcription Factor 1
FAS	Fatty Acid Synthase
HMGCR	3-hydroxy-3-methylglutaryl-CoA reductase
RCT	Reverse Cholesterol Transport
LXR	Liver X Receptor
RXR	Retinoid X Receptor
PGC-1α	Proliferator-Activated Receptor Gamma Coactivator 1-alpha
HDAC	histone deacetylases
HATs	histone acetyltransferase
lncRNAs	long non-coding RNAs
ceRNA	competitive endogenous RNA
LPS	lipopolysaccharide
IFN-γ	interferon-γ
STAT1	Activator of Transcription 1
IRF5	Interferon Regulatory Factor 5
iNOS	inducible nitric oxide synthase
NO	nitric oxide
ROS	reactive oxygen species
Arg1	arginase 1
TGF-β	transforming growth factor-β
HSCs	hematopoietic stem cells
GMPs	granulocyte-monocyte progenitors
BMDMs	bone marrow-derived macrophages
AP-1	activator protein 1
SOCS1	cytokine signaling 1
SHIP1	SH2 domain-containing inositol 5′-phosphatase 1
TAB2	TAK1-binding protein 2
IKKε	nuclear factor-κB kinase subunit ε
NIK	NF-κB-inducing kinase
IRAK1	interleukin-1 receptor-associated kinase 1
TRAF6	TNF receptor-associated factor 6
MyD88	myeloid differentiation primary response 88
JNK	c-Jun N-terminal kinases
ERK	extracellular signal-regulated kinases
ASK1	Apoptotic signal-regulating kinase 1
ECs	endothelial cells
EGF	Epidermal Growth Factor
JAKs	receptor-associated Janus kinases
SH2	Src homology 2
LRP6	lipoprotein receptor-related protein 6
TBL1X	transducin β-like 1 X-linked
TREM2	triggering receptor expressed on myeloid cells 2
PIK3CA	PI3K catalytic subunit α
Beclin-1	Beclin autophagy-related 1
LC3-II	microtubule-associated protein 1A/1B-light chain 3-II
Mrc-1	mannose receptor
PPARγ	Peroxisome proliferator-activated receptor γ
ERK1/2	extracellular signal-regulated kinase 1/2
KLF	Krüppel-like factor
SIRT1	Sirtuin 1
scRNA-seq	single-cell RNA sequencing
Nrf2	nuclear factor erythroid 2-related factor 2
LPL	lipoprotein lipase
ULK1	Unc-51 Like Autophagy Activating Kinase 1
ATG5	Autophagy-related 5
TIMI	Thrombolysis in Myocardial Infarction
AUC	Area Under the Curve
CAD	coronary artery disease
ROC	Receiver Operating Characteristic
EVs	extracellular vesicles
ACS	acute coronary syndrome
LAMs	lipid-associated macrophages
MHCII	major histocompatibility complex class II

## CRediT authorship contribution statement

**Fan Fan:** Writing – original draft, Methodology. **De-Jing Shang:** Writing – review & editing, Supervision, Conceptualization.

## Availability of data and materials

The datasets generated and/or analyzed during the current study are available from the corresponding author upon reasonable request.

## Ethics approval and consent to participate

All animal maintenance and procedures were performed in accordance with the recommendations established by the Animal Care and Ethics Committee of Liaoning Normal University following the Basel Declaration. Ethics Approval was granted by the Animal Care and Ethics Committee of Liaoning Normal University. No human subjects.

## Funding

This work was supported by the 10.13039/501100001809National Natural Science Foundation of China [grant numbers 32070440, 32270513, 32170499 and 31702023] and the Central Government-guided Local Scientific and Technological Development Fund.

## Declaration of competing interest

The authors declare that they have no competing interests.
